# Auto-segmentation for total marrow irradiation

**DOI:** 10.3389/fonc.2022.970425

**Published:** 2022-08-30

**Authors:** William Tyler Watkins, Kun Qing, Chunhui Han, Susanta Hui, An Liu

**Affiliations:** Department of Radiation Oncology, City of Hope National Medical Center, Duarte, CA, United States

**Keywords:** auto-segmentation, auto-contouring, artificial intelligence, total marrow irradiation, total marrow lymphoid irradiation

## Abstract

**Purpose:**

To evaluate the accuracy and efficiency of Artificial-Intelligence (AI) segmentation in Total Marrow Irradiation (TMI) including contours throughout the head and neck (H&N), thorax, abdomen, and pelvis.

**Methods:**

An AI segmentation software was clinically introduced for total body contouring in TMI including 27 organs at risk (OARs) and 4 planning target volumes (PTVs). This work compares the clinically utilized contours to the AI-TMI contours for 21 patients. Structure and image dicom data was used to generate comparisons including volumetric, spatial, and dosimetric variations between the AI- and human-edited contour sets. Conventional volume and surface measures including the Sørensen–Dice coefficient (Dice) and the 95^th^% Hausdorff Distance (HD95) were used, and novel efficiency metrics were introduced. The clinical efficiency gains were estimated by the percentage of the AI-contour-surface within 1mm of the clinical contour surface. An unedited AI-contour has an efficiency gain=100%, an AI-contour with 70% of its surface<1mm from a clinical contour has an efficiency gain of 70%. The dosimetric deviations were estimated from the clinical dose distribution to compute the dose volume histogram (DVH) for all structures.

**Results:**

A total of 467 contours were compared in the 21 patients. In PTVs, contour surfaces deviated by >1mm in 38.6% ± 23.1% of structures, an average efficiency gain of 61.4%. Deviations >5mm were detected in 12.0% ± 21.3% of the PTV contours. In OARs, deviations >1mm were detected in 24.4% ± 27.1% of the structure surfaces and >5mm in 7.2% ± 18.0%; an average clinical efficiency gain of 75.6%. In H&N OARs, efficiency gains ranged from 42% in optic chiasm to 100% in eyes (unedited in all cases). In thorax, average efficiency gains were >80% in spinal cord, heart, and both lungs. Efficiency gains ranged from 60-70% in spleen, stomach, rectum, and bowel and 75-84% in liver, kidney, and bladder. DVH differences exceeded 0.05 in 109/467 curves at any dose level. The most common 5%-DVH variations were in esophagus (86%), rectum (48%), and PTVs (22%).

**Conclusions:**

AI auto-segmentation software offers a powerful solution for enhanced efficiency in TMI treatment planning. Whole body segmentation including PTVs and normal organs was successful based on spatial and dosimetric comparison.

## 1 Introduction

Segmentation of human anatomy on medical images is a critical component of targeted radiation therapy (RT). Delineations are used to design radiation therapy treatment plans including conformal avoidance in 3D-conformal RT (3DCRT) and as input to optimization algorithms for intensity modulated radiation therapy (IMRT). These delineations are typically performed by clinicians utilizing manual contouring software, which allows for drawing structures on medical images and tools including smoothing, interpolation, and intensity-based thresholding. The accuracy of the delineation is perceived as a critical element of modern IMRT and currently serves as a safety mechanism for monitoring dose to organs at risk (OARs). The 3D-dose distribution is evaluated using a 2-dimensional dose volume histogram (DVH) of proximal OARs and targets, and the results of many studies reports “safe” DVH levels for OARs based on clinical trials and clinical experience to guide future treatments. The process of manual segmentation, DVH evaluation, and multiple layers of human review (dosimetrists, physicians, and physicists) has allowed for the successful introduction of high-precision IMRT, including total marrow irradiation (TMI) and total marrow and lymphoid irradiation (TMLI) (1–3). TMI and TMLI treatment planning requires extensive contouring but allows enhancement the antileukemic effect by delivering higher doses to the region of leukemia niche, the bone marrow and lymph nodes, while reducing organ dose exposure compared to conventional total body irradiation (TBI). TMLI significantly improved overall survival in patient with relapse/refractory leukemia when compared to TBI (4). Dose escalation using conventional TBI did not improve survival because of radiation-induced toxicities (5). While evidence of the clinical advantages of TMI is growing, clinical implementation will rely on the technological capabilities of adopting institutions. The significant manual effort required in contouring the entire body including OARs, and TMLI target volumes is a major barrier to clinical implementation.

The potential to replace human delineation with computerized methods has been a focus of image science for decades. The continued interest in Artificial Intelligence (AI) for this manual task is predicated on the accuracy, consistency, and human trust in the software. However, there are not universally accepted methods to determine whether segmentation is accurate, precise, or reliable in human- or algorithm- defined delineations. Despite significant efforts in computer vision and shape modeling including atlas-based methods (6, 7), deformable-image registration (DIR) (8–10), probabilistic modeling (11), and AI, fully automated segmentation remains infeasible. None of these advancements have broken through to consistently replace manual delineation in RT (12, 13) despite retrospective evidence that auto-contouring may be more consistent in estimating DVH dose associated risk (14). In order to validate auto-segmentation for clinical use, tools include human scoring (12, 15) and computational indices including the Sørensen-Dice coefficient (Dice) and the Hausdorff Distance (HD) have consistently been deployed, but they provide limited value in measuring clinical efficiency gains.

The perceived critical importance of contour accuracy has led to considerable time and effort in manual review and manual edits of automated contours produced by these algorithms, and limited efficiency gains. More recently, AI- deep and transfer learning methods are being used for auto-segmentation with significant promise for clinical adoption due to their accuracy and consistency despite the lack of widely accepted criteria for defining accuracy and consistency. Several recent studies report on head-and-neck (H&N) and pelvis auto-segmentation have been evaluated using Dice, HD and other metrics (16). Dice and HD were also used to evaluate the H&N Auto-Segmentation Challenge 2015 (17). The common approach of the reviewed studies is three-fold, (1) select sets of comparison metrics, (2) generate auto-segmentations, and (3) compare auto-segmentations with clinical/database contours. This approach has been published in anatomic sub-sites including H&N (16, 18), thorax (19, 20), abdomen (21, 22), pelvis (23–25), and whole body (26). Unlike these studies, our institution has clinically introduced an AI- auto-segmentation software, Medical Mind, Inc. (27) for all RT patients. All patient images are auto segmented prior to dosimetrist and physician interaction in the treatment planning system. The Medical Mind software has been clinically deployed for all normal contours in external beam treatment planning including brain, H&N, thorax, abdomen, and pelvic RT. Using this approach, this work demonstrates prospective congruence between AI- and clinical segmentations.

As an experienced innovator in TMI/TMLI treatment, a TMI/TMLI AI-contouring model was developed based on clinical data from patients treated at our institution. By providing approximately 100 prior clinical cases of total body contouring from TMI/TMLI treatments including planning target volumes (PTVs), a TMI/TMLI AI-contouring model was optimized using the Medical Mind software. The TMI/TMLI model includes 27 individual OARs and 4 PTVs to assist with the laborious task of total body contouring and to create consistency in the clinical treatment planning workflow. The TMI/TMLI PTVs are based on normal anatomy (not tumors) and therefore can potentially be reliably, automatically identified. The OAR set includes important regions of brain, H&N, thorax, abdomen, pelvis, and extremities in a single set of contours. In the current workflow, clinicians including dosimetrists, physicians, and physicists are presented with the auto-segmentations prior to human delineation. In this method, the clinician must make a clinical judgement about whether an AI- contour edit is important and necessary to clinical treatment plans. This work describes the initial experience and success of the TMI/TMLI contouring model in a prospective approach.

## 2 Methods

The Medical Mind AI-software was trained on 100 prior clinical TMLI clinical patients including OARs and PTVs. The 100 TMI patients were treated consecutively at our institution. The datasets and contours include multiple dosimetrist, physicist, and physician contributions and standard planning guidelines were used to ensure consistency. The model was implemented using Python 3.6 (28) and PyTorch 1.0 (29). The dataset was split into a training set, a validation set, and the testing set. The training set contained 70 patients, the validation set contained 15 patients and the testing set also contained 15 patients. The Medical Mind software uses a Convolutional Neural Network (CNN) following the U-Net architecture. It contains an encoder and a decoder, and the convolutional layers are replaced by context aggregation blocks. Both the encoder and decoder consist of five context aggregation blocks. The feature maps in the encoder part are concatenated to the corresponding feature map in the decoder part. The Adam optimization algorithm (30) was used with a 0.001 learning rate. The model was trained over 50 epochs and the best model was selected, which is the one that had the lowest validation loss score. The convolutional layers were initialized using Xavier Uniform Initialization (31). All these convolution layers were followed by a batch normalization layer and a Rectified Linear Unit (ReLU) layer. The model was trained and tested using a GTX 1660-S GPU.

The model was deployed clinically, and this work details our initial (6-month) experience with the TMI/TMLI patients using the system. This work compares the final, clinically approved and the original AI- contours through volume, surface, and composite comparison metrics. The edited and original AI contour dosimetry evaluated on the planning dose are also compared and correlated to the various comparison metrics. Efficiency gains are estimated based on the relative number of manual edits performed on the AI-contours.

Computed Tomography (CT) simulation for TMI/TMLI treatment planning is acquired in a head-first supine (HFS) position spanning head-to-toes. The HFS images are sent from CT-simulation to the Medical Mind software, auto-segmentation is performed by selecting the TMI/TMLI model, delineating, and sending for import into treatment planning software. The process of opening the Medical Mind software *via* a secure interface to an on-network PC, identifying the patient, auto-segmentation of the patient, sending to clinical treatment planning software, and importing the AI-contours is typically<7 min. For each patient, at least one dosimetrist and at least one radiation oncologist review the AI-segmentations and edit PTVs and OARs prior to clinical treatment planning. The HFS CT is split at mid-thigh and flipped to FFS for treatment delivery from head to mid-thigh in HFS, and from the toes to mid-thigh in FFS, with composite dosimetry in the junction evaluated at the time of treatment planning. Contours and dosimetry were evaluated only on the HFS scan. In the FFS scan, only PTV-bone is included and delivery is often simple parallel-opposed beams.

**Figure 1:**
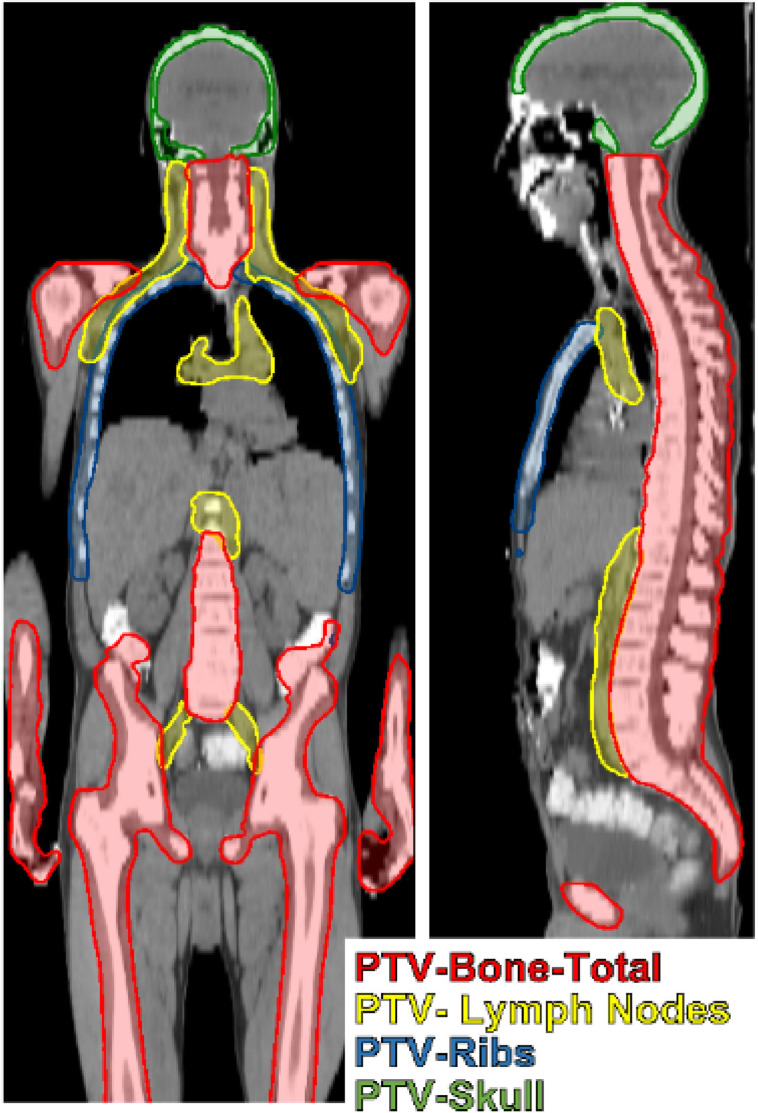
TMI/TMLI target volumes include total bone with margin (PTV-Bone), lymph nodes extending from the head and neck to the pelvis with margin (PTV-Lymph Nodes), the chestwall containing ribs and the mediastinum (PTV-ribs), and the skull (PTV-skull).

The Medical Mind TMI-model generates 21 unique OARs (plus 6-additional Left/Right pairs) and 4 PTVs. The OARs are detailed in **Table 1**. The four PTVs are the PTV-Bone, the PTV- lymph nodes (PTV-LNs), PTV-ribs, and PTV-skull. The PTVs are 1-10 mm expansions of anatomic structures visible on CT and were trained on clinically utilized PTVs from the 100 prior TMI patients. The PTV-Bone is approximately an 8 mm expansion of all bone excluding skull and rib. The PTV-skull is an approximate 1-mm expansion of the skull. PTV-LNs includes approximately 5 mm margins about cervical, axillary, mediastinal, paraaortic, and pelvic lymph nodes. The PTV-ribs includes the chest wall, all ribs, and abuts the spinal canal. **Figure 1** shows an example PTV set generated from the Medical Mind TMI/TMLI model.

**Table 1:** Organ at Risk (OAR) contours included in the TMI/TMLI auto-segmentation model.

H&N		Thorax	Abdomen	Pelvis
Brain	Mandible	Esophagus	Spleen	Bowel Bag
Eyes (L+R)	Oral Cavity	Heart	Stomach	Bladder
Lens (L+R)	Larynx	Lungs (L+R)	Liver	Rectum
Optic Nerve (L+R)	Thyroid	Spinal Cord	Kidneys (L+R)	
Optic Chiasm	Parotids (L+R)			

Since introducing the auto-segmentation TMLI-model and compilation of data, 21 patients were treated with this workflow. All CT scans were performed with 7.5 mm slice spacing (range of 134-262 slices per patient), and in-plan voxel resolution ranged from 0.98-1.56 mm. Treatment Delivery was designed to deliver dose to bone, bone marrow, blood, and lymphoid tissue. Treatment planning has been previously examined in detail including early development on Tomotherapy (Accuray, Inc) for helical delivery (1) and on Eclipse for multi-iso volumetric modulated arc therapy (VMAT) (32). Eighteen of the patients were treated with helical Tomotherapy, three were treated with multi-isocenter VMAT on the Varian Truebeam (Varian Medical Systems, Palo Alto, CA) linear accelerator.

### 2.1 Region of interest comparison metrics

Common metrics to compare AI- and edited segmentations include volume and surface overlap measures. Volume overlap methods include the Sørensen-Dice coefficient (Dice). Dice presents the relative overlap of two segmentations, where a value of 0 is no overlap and a value of 1 is 100% overlapped. However, Dice can be very misleading in terms of segmentation quality. For example, structures with large volumes can have a high-volume overlap (Dice > 90%) with possibly 100% of the surface deviating by a large distance. For 2-spheres of radius r1 and r2, dice is >0.90 for all r1/r2 within 3%, which is >3mm for all r1, r2>10cm. Dice is an important measure of contour quality and overlap, but perhaps not a sufficient measure of contour quality or efficiency.

Surface metrics to measure contour accuracy include the Hausdorff Distance (HD) (33). Measured at each point of the surface, the HD creates a distribution of Euclidean distances to nearest points. HD is typically expressed as a percentile; the 95th percentile (HD95) presents the maximum distance from surface-to-surface for 95% of the reference to test contour surface. An HD95 = 5 mm demonstrates 95% of the surface points are less than 5 mm. However, presenting HD at a percentile does not provide adequate information to assess clinical contour quality or efficiency gains. For example, an HD95 = 3.1mm could imply 100% of the contour deviates by >3mm or could imply 94.9% has HD<1mm and 5.1% deviates by >3mm. Like Dice, HD95 offers value but is not an adequate assessment of contour quality or efficiency gains.

Due to the limitations in classical volume overlap including Dice and HD percentiles (for example HD95) for estimating contour quality and efficiency gains, we aim to measure the congruence of the AI- and human- contour by the relative surface which deviates by less than a fixed distance *δ*. This can be presented as the fraction or the percent of the surface which deviates by less than this distance. For segmentations defined by the set of 3D-points X and Y with elements xi and yi, the HD can be written


$$\;HD\left(X,Y\right)=\max\left\{\sup_{\text{xi}\in \text{X}}inf\;\left(xi,\;Y\right),\sup_{\text{yi}\in \text{Y}}\;inf\left(X,yi\right)\right\}$$

Where sup is the least upper bound or supremum, inf is the greatest upper bound or the infimum. We define the efficiency gain (*Eff*) at a spatial tolerance *δ* by the ratio of the cardinality (card) of the sets HD(X,Y)<*δ* and HD(X,Y):


$$Eff\left(\delta \right)=\frac{card\left(HD<\delta \right)}{card\left(HD\right)}\;$$

Where it is assumed, all elements are unique since each represents a unique spatial position on the surface. We propose the efficiency measure evaluated at distance *δ* =1mm, and efficiency is the relative contour surface with HD<1mm. The 1mm distance is approximately equal to the axial intra-voxel spacing of the image and therefore can be considered the relative amount of the AI-contour which was edited by the clinician, and an unedited contour is related to enhanced efficiency. The clinical efficiency gain was estimated by the percentage of the AI-contour-surface within 1mm of the clinical contour surface. Examples of efficiency gains include unedited AI-contours will have HD<1mm = 100% and an efficiency gain of 100%, an AI-contour with HD<1mm = 70% has an efficiency gain of 70%.

Computation of Dice and HD was performed in MATLAB using binary images defined on the CT-coordinates. Sub-voxel vertices were not considered. Dice is a built-in function of MATLAB (34) and was computed on AI and human edited AI contours for all OARs and PTVs. HD was computed as the Euclidean distance arrays between voxels of the binary images *via* the distance transform function in MATLAB (35). In this analysis, Dice, HD<1mm, and HD95 each provide unique information about overall contour quality (Dice volume overlap), the potential efficiency gains (HD<1mm), and the magnitude of the surface deviations (HD95). Section 3.1 details contour similarity metrics in the patient dataset.

### 2.2 Dose volume histogram comparison

All treatments were delivered twice-daily (BID) at PTV dose levels including 12 Gy in 8 fractions, 14 Gy in 8 fractions, 18 Gy in 9 fractions, and 20 Gy in 10 fractions. Doses varied based on protocol and patient, but dosimetry goals in the PTVs consistently included the volume which receives 100% of prescription dose was > 85% (V100%>85%). DVH planning objectives for OARs followed institutional protocols (36) including dose to 10% volume (D10), dose to 50% volume (D50), and dose to 80% volume (D80) with levels specified from population statistics of various initial testing and patient cases. Lung mean dose was limited to 8 Gy in all cases, kidney dose was not limited consistently. The clinical dose distribution of the upper body plans includes H&N, thorax, abdomen, and pelvic regions. This clinical dose distribution was used to computed cumulative DVH based on sampling both clinical and AI- contours. Plans were not re-optimized on each contour set. The DVHs were compared in order to estimate the potential dosimetric significance of contour error.

The cumulative DVH was computed for all AS- and clinical- contours utilizing the dicompyler python module (37). DVH calculations were performed at the axial voxel resolutions (0.98-1.56 mm) and three dose and ROI slices per 7.5 mm CT-slice resulted in a longitudinal resolution of 2.5 mm. DVH was computed for each ROI, and DVH-differences were estimated at all dose levels for all structures in 1 cGy dosimetric bins. Using a relative volume difference of 0.05 at any dose level to flag a potentially clinically significant DVH difference, then DVH was compared along neighboring dose levels using a dose tolerance threshold of 20 cGy. All DVH differences which exceed 0.05 relative volume difference at dose levels ≥20cGy were flagged as “failing” criteria.

To compare the distributions of Dice, HD95, and HD<1mm between “passing” and “failing” structure DVH sets, the two-sample Kolmogorov-Smirnov (KS) test was used as implemented in MATLAB. The 2-sample KS-test assumes continuous distributions, and significance testing was performed at a 5% significance level. In order to determine which contour metric (Dice, HD95, and HD<1mm) best predicts DVH variations, the KS test statistic was used to measure distance between the distributions. Section 3.2 summarizes the observed dosimetric differences and correlations them with Dice, HD95, and HD<1mm.

## 3 Results

### 3.1 Region of interest comparisons

A total of 467 contours were compared in the 21 patients. In estimates from clinical staff, contouring time for complete contouring of TMI/TMLI cases was reduced from 4-8 hours for full manual contouring to 1-3 hours by editing the AI- contours, or roughly a 75% efficiency gain. These efficiency gains were directly reflected in high rates of unedited contours estimated from HD<1mm. In all OARs, deviations >1mm were detected in 24.4% ± 27.1% of the structure surfaces; an average clinical efficiency gain of 75.6%. Deviations > 5mm were detected in 7.2% ± 18.0% of all OAR contours. The efficiency metric was not well correlated to DICE (r = 0.76) or negatively correlated to HD95 (r=-0.52) indicating the efficiency gains from AI-contouring is not trivially related to these traditional overlap or surface metrics.

**Figure 2:**
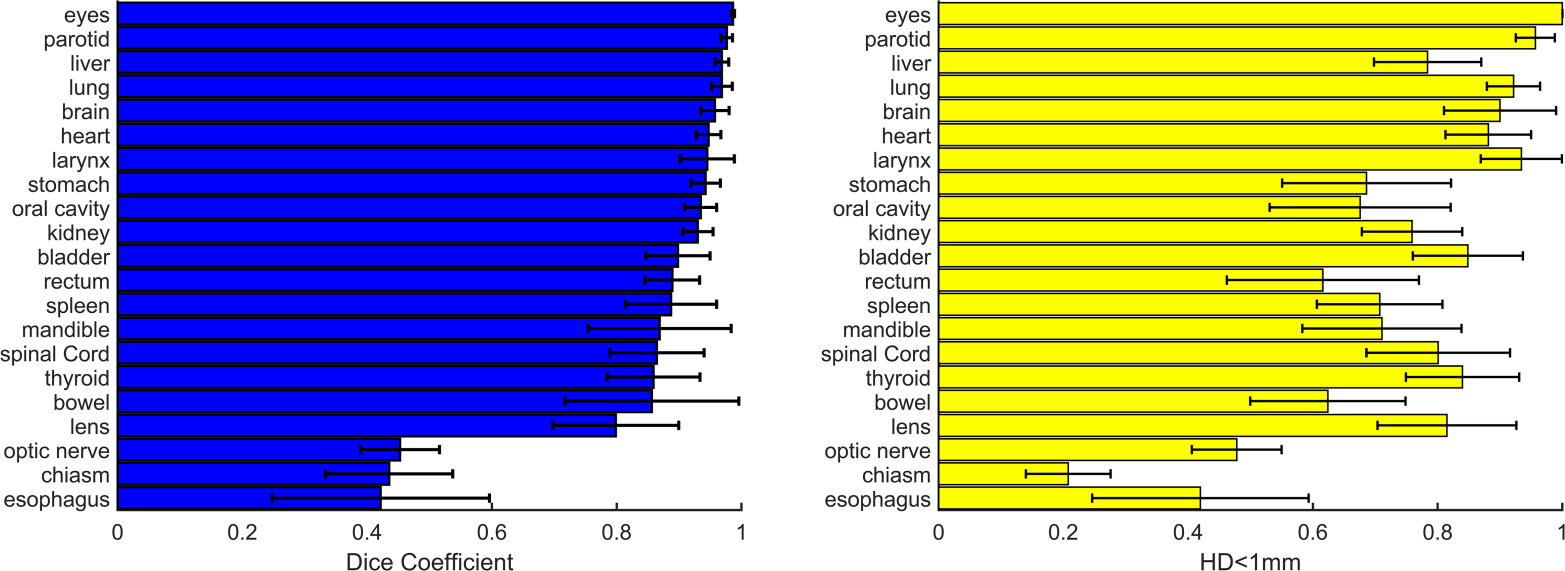
Dice (left) and relative contour surface with HD<1mm (right) for all structures. Standard deviations for the patient population for each structure are shown. In 18/21 contours average Dice is >0.8. Efficiency gains estimated from HD<1mm are >0.60 in 18/21 structures.

**Figure 2** shows dice and HD<1mm for all OAR structures (right/left pairs are grouped). Of the 21 structures, 18/21 have Dice>0.8, with esophagus, optic nerves, and optic chiasm with significantly lower Dice coefficients. As shown in **Figure 2**, average Dice > 0.9 was observed in relatively large structures including stomach, oral cavity, rectum, and bowel but average HD<1mm was<0.7 in these cases.

In H&N OARs, efficiency gains ranged from a low of 42% in optic chiasm to 100% in eyes (unedited in all cases), with mean and standard deviation 77.3% ± 18.9%. HD95 was >1mm in only oral cavity (1.02mm). The optic nerves and chiasm were edited significantly for all patients, but this may be a function of large slice spacing used in TMI/TMLI CT-simulation, in general the clinical contours were larger than the AI-contours. The HD95 was not well correlated to HD<1mm (r=-0.34) in the H&N area.

In thorax OARs, average efficiency gains were >80% in spinal cord, heart, and both lungs, with average esophagus HD<1mm just 21%. HD95 was ≤1 mm in lungs and spinal cord, heart, and spinal cord. Differences in lung and heart were visually evident in structures with Dice< 0.9 and in at least one case, were due human error due to window/leveling variations in borders. Differences in spinal cord were evident but minimal. The esophagus consistently scored low dice, low efficiency, and relatively high HD95. The AI-segmented esophagus did not extend superiorly into the cricoid cartilage border, instead including just a portion of the esophagus in the T-spine region, leading to significant human edits.

In abdomen and pelvis OARs, efficiency gains ranged from 60-70% in spleen, stomach, rectum, and bowel and 75-84% in liver, kidney, and bladder. These results were associated with relatively high values of HD95 indicating clinically significant edits in spleen (2.1 ± 4.0 mm), rectum (2.7 ± 3.2 mm), and bowel (2.5 ± 2.8 mm). Bowel was dependent on edits to include the entire bowel bag, or individual bowel loops. Similar to esophagus, the kidneys consistently showed human edits to correct the superior border of the AI-contours. Average HD<1mm was 75.8%, and HD95 was 1.4 ± 1.6 mm. However, in the (approximately) 25% of the non-overlapping, superior region of kidney was HD95 was 8.7 mm. In this case, manually adding slices to the superior kidney contour can still represent a significant clinical efficiency gain even though metrics indicate edits could be significant.

Results in PTVs were generally worse than in OARs but still show significant efficiency gains in 4 PTVs. All PTV data is shown in **Table 2**. Average Dice across all patients and PTVs was 86% ± 15%, HD<1mm was 61% ± 23%, and HD95 was 11.0 ± 22.2 mm.

**Table 2:** PTV statistics are summarized including Dice, relative surface with Hausdorff Distance<1mm (HD<1mm), and the 95th percentile of HD.

PTV	PTV-Bone	PTV-Lymph nodes	PTV-Ribs	PTV-Skull
Dice	85.1% ± 21.9%	83.0% ± 16.6%	94.6% ± 4.4%	81.4% ± 9.9%
HD<1mm	40.6% ± 22.1%	53.4% ± 12.8%	80.1% ± 11.7%	72.4% ± 20.0%
HD95	30.5 mm ± 38.0mm	7.5mm ± 3.6mm	3.0mm ± 2.6mm	2.8mm ± 2.2mm

**Figure 3:**
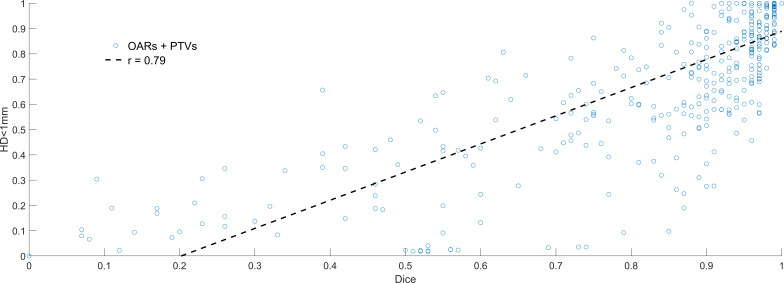
Dice (x-axis) vs. relative surface area with Hausdorff Distance<1mm (HD<1mm). Low Dice values (<0.5) equated to low HD<1mm in almost all cases, but the converse was not true. High Dice (>0.8) could still result in significant human editing, with HD<1mm ranging from 0.1-0.9 in this region.

**Figure 4:**
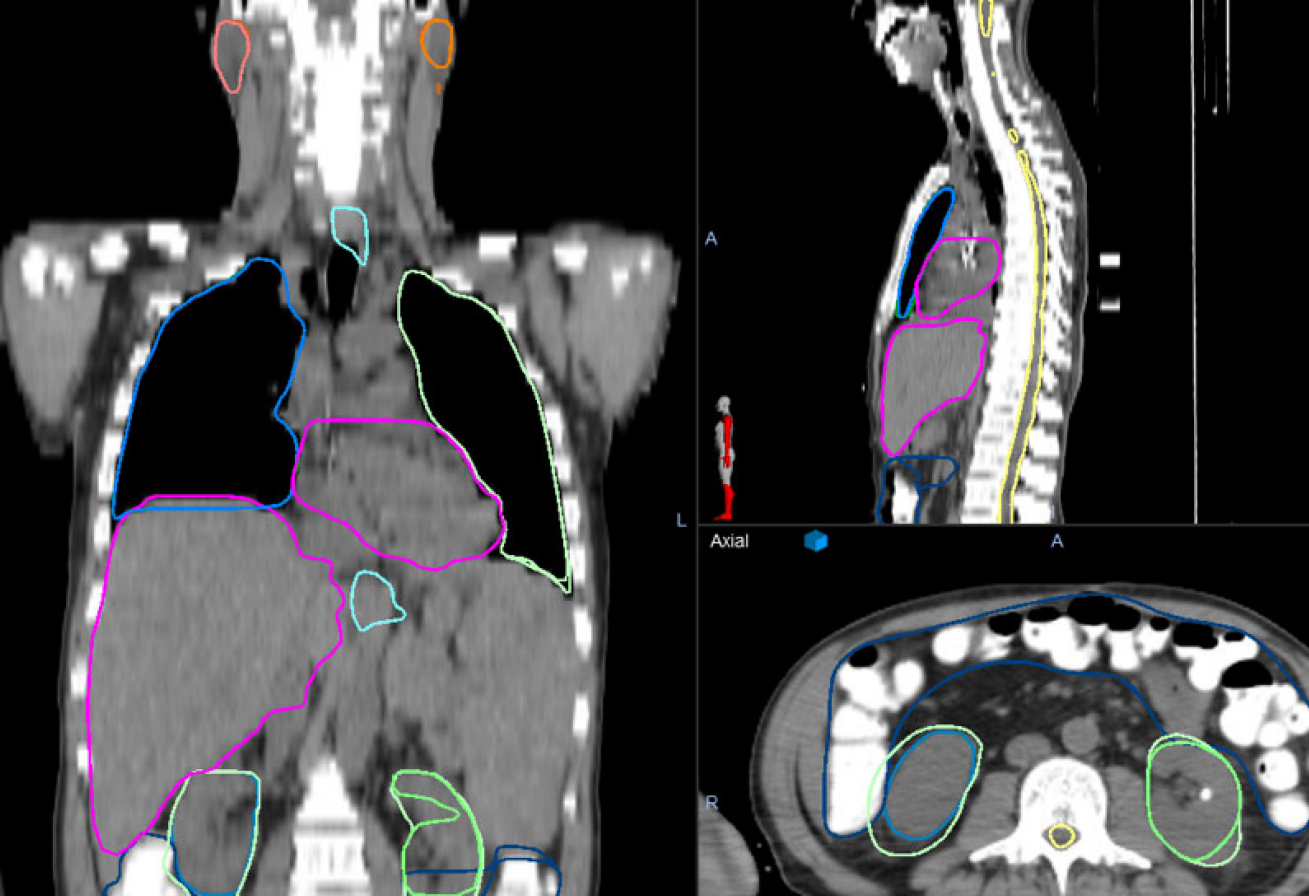
An example patient image is shown including manual and AI contours in parotids, larynx, lung, heart, liver, and kidneys. The human and TMI contours are indistinguishable in most organs, however the kidneys demonstrate some variation In the axial image (borrom right) the human drawn kidneys are significantly larger than the AI-kidneys due to clinician preference.

There was a weak correlation between Dice and HD<1mm (r=0.79). Dice values >0.8 did not imply high values of HD<1mm, demonstrating that structures can have significant overlap with potentially meaningful surface disagreement. **Figure 3** shows HD<1mm as a function of Dice. In general, only a very high Dice value (>0.98) ensures and HD<1mm is >0.7. Correlations between HD<1mm and HD95 were not strong (r=-0.65). This weak correlation is expected, HD95 describes the largest discrepancy, HD<1mm is a very stringent metric demonstrating overlapping surfaces. An example patient image including manual and AI contours is shown in **Figure 4**.

### 3.2 Dose volume histogram comparisons

DVH differences exceeded 5% relative volume difference in 109/467 of all curves at any dose level. The most common 5%-DVH variations were in esophagus (86%), rectum (48%), and PTVS (22%).

**Figure 5:**
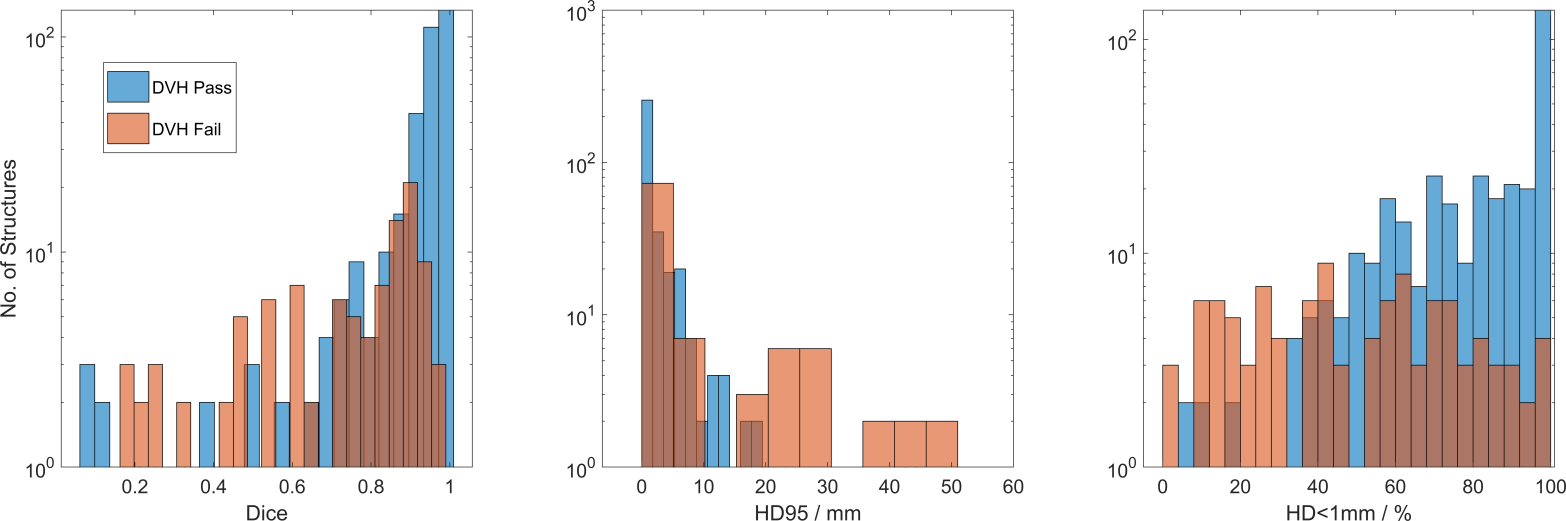
Dice, HD95, and HD<1mm cases which pass (blue) and fail (red) the applied DVH criteria. Low Dice and low HD<1mm still showed similar DVH in structures which received low, or no dose. HD95>20mm showed no passing DVH.

Dice, HD95, and HD<1mm were statistically different between the pass and fail groups (*p*<10^-7^ in all comparisons). The KS-statistic measures distance between the distributions and indicates the Dice KS-statistic (0.65) was more predictive than HD95 (0.37) or HD<1mm (0.48) in predicating DVH differences. The average Dice in passing vs. failing DVH was 0.91 ± 0.16 vs. 0.71 ± 0.24. Average HD95 was 2.2 ± 4.6 mm vs. 11.9 ± 22.6 mm and HD<1mm was 80% ± 22% vs. 51% ± 27%. All values show a clear distinction between passing and failing DVH-criteria groups, and the distributions are shown in **Figure 5**. This result is intuitive since Dice and DVH are volume-based metrics whereas HD is a surface metric.

The obvious result that DVH metrics will fail with low DICE is not thought-provoking. In 15 cases where low Dice score passed the DVH criteria of 0.05 relative volume/20cGy tolerance level, 6 were in optic chiasm and 5 were in optic nerves. This demonstrates limitations cumulative DVH, as significant dose-volume differences can be hidden by normalizing to relative volume even in high-gradient areas. Another source of false-positive DVH match is uniform dose; any 2 structures will have equal cumulative DVH in a uniform dose area. In lens, 6/21 cases failed DVH criteria, with 2/21 cases showing major differences in maximum dose between clinical contours (Dmax<3.5 Gy) and AI-contours (>5 Gy). In optic structures including chiasm and nerves, 6/63 cases failed the DVH criteria with Dmax differences >2 Gy in 3 cases.

There is possibly a lower limit on usable structures in dose and DVH calculation from AI-contours (where Dice<0.6), but there also exists a grey area in Dice correlation where the pass/fail distributions significantly overlap in the range 0.7-0.98. Structures which failed DVH-criteria with Dice in the range of 0.7-0.98 include 13 PTVs and 56 OARs. PTVs which failed DVH-criteria with Dice>0.7 show the AI-DVH is lower than the human contour DVH, indicating AI-contours outside of the clinically used volumes. Over-contouring of PTVs, if used in planning, would result in high dose to normal, untargeted tissues. Despite relatively high DICE scores (>0.93) in six cases, DVH differences exceeded 0.30 relative volume at all doses in many cases. These results demonstrate the critical importance of PTV contouring in highly conformal radiation such as TMI/TMLI.

**Figure 6:**
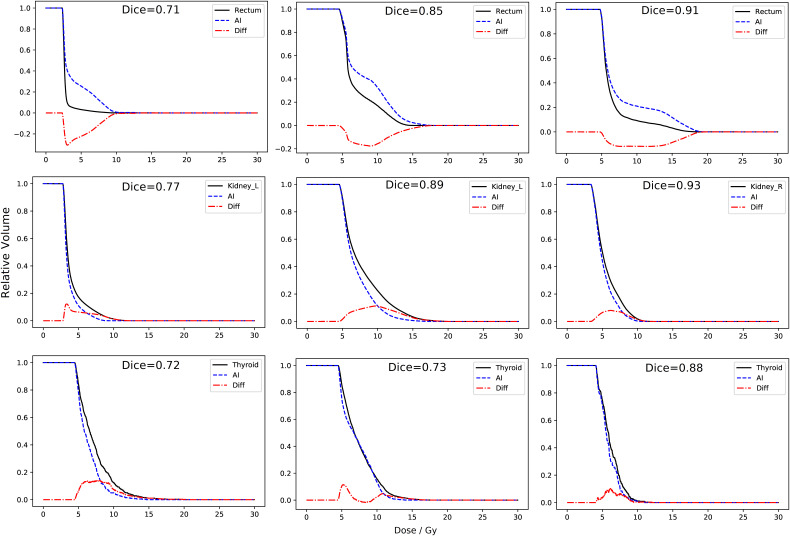
DVHs computed from Human (solid) and AI- (dashed) contours, with difference. (top) AI-rectum contours were > human DVH in 8/9 cases. (middle) Kidney AI-contours of kidney resulted in DVHs consistently lower than the human contours. Thyroid showed minor differences independent of relatively low Dice values.

There were 56 OARs which failed DVH-criteria with Dice>0.7 including 6 brain contours, 5 thyroid contours, 6 kidney contours, and 9 rectum contours. Consistent DVH differences were observed for the same OARs across many patients. In the case of Rectum, 8/9 AI-DVHs were > human-DVHs due to humans adding regions of sigmoid bowel to the rectum contour. In the case of Kidney, missing regions of superior kidney resulted in AI-DVHs< human DVHs in all cases. **Figure 6** shows DVH computed on AI and human contours for (top) 3 rectum cases. Dice ranges from 0.71 (left) to 0.91 (right), but the DVH differences are not significantly reduced as Dice increases. Similar results were observed in Kidney (middle) and Thyroid (bottom) with Dice ranging from 0.77 to 0.93. There was not a significant reduction in DVH differences as Dice is increased from 0.7-0.9 in these structures.

## 4 Discussion

The TMI model was trained on >100 patients and validated in the current study on 21 patients. This modest sample size showed excellent results in most structures in all patients, but it is possible additional patients may reveal additional problems with the AI-segmentations. AI-models from trained from limited data are known to be susceptible to overfitting, including in auto-segmentation (38). In this sample, we have shown the Medical Mind TMI/TMLI model is consistent and reliable for contouring a vast majority of structures. We show significant DVH differences in high gradient areas (PTVs and optic structures) which reinforces our current workflow of AI-contouring followed by manual review. However, for many OARs, even low Dice overlap may not result in significant errors in DVH estimates and may be reliable to use clinically without edits.

The clinical validity of AI-defined OARs evaluated by Dice and HD95 has been assumed in prostate (39) including in physician-edited contours in a prospective study (40). The current study has demonstrated limited value in Dice and HD95, and therefore introduces a more stringent comparison metric, HD<1mm. HD<1mm is the relative proportion of contour surface within 1mm of the clinical contour. In prospective studies where AI-contours are used as a starting point for clinical contours, we strongly recommend using this or a similar, more challenging metric in describing the accuracy of the segmentation.

In DVH calculation, we found Dice is the strongest predictor of DVH congruence. However, a high Dice value did not ensure DVH differences<0.05, and DVH for structures with Dice >0.9 was not significantly different than structures with dice in the range of 0.7-0.8.

The TMI/TMLI dataset is unique for its use of relatively large slice spacing (7.5 mm). However, from a clinical perspective this is equivalent to defining contours on every other 2.5mm slice and utilizing interpolation. DVH calculation was performed on a 2.5mm grid, so that large axial contour variations would not heavily influence dose evaluation. Medical physics datasets including H&N MRI (18) and thoracic cancers (19, 20), rectal cancers (41), and cervical cancers (25, 42) have been made available to validate and inter-compare auto-segmentation algorithms. The TMI/TMLI experience at our institution can contribute to this meaningful inter-institution comparison of AI algorithms for total body contouring. This is the first study demonstrating AI-segmentation in the whole body simultaneously. Chen et al. (26) introduced an AI-algorithm called “WBNet” and achieved average Dice in the range of 0.81-0.84 in a large number of datasets including H&N, thorax, abdomen, and pelvis sites individually. In our comparisons, only esophagus, lens, optic nerves, and optic chiasm showed average Dice<0.85. These impressive results were realized in terms of clinical efficiency gains as well, with team members routinely reporting 50-90% efficiency gains in contouring these complex cases.

In small structures such as lens and optic nerves and chiasm, a low Dice score does not imply a poor contour. Due to their limited size, a very small deviation can lead to a very low Dice score. A known limitation of multi-layer deep learning in image recognition and segmentation is limited number of features (43–45) which may explain why optic nerves, and chiasm are among the worst scoring structures in Dice in the current study. However, average Dice 0.42-0.45 in the optic structures are not significantly lower than those reported in other studies, 0.37-0.65 (46) and 0.45-0.69 (47). In these small structures, a single 7.5-mm slice difference in the TMI/TMLI contour set can lead to large deviations. From a clinical efficiency perspective, these relatively small structures (<1cc volume) are defined on as few as 1 TMLI slice and do not add significant workload to manual contouring when compared to larger structures like brain, lung, and liver which require contouring on dozens of CT-slices. In esophagus, boundary errors led to low DSC and high HD95. Esophagus needs closer scrutiny; it’s known to potentially include large inter-slice positional variations and low CT-contrast. In optic structures, esophagus, and similar structures which can vary significantly over small regions of cranio-caudal anatomy, training AI on large slice spacing images may lead to significant errors if applied to finer resolution images.

It may be possible to link AI algorithms with contour quality assurance using, for example a multi-parametric approach (15, 48) or machine learning approach (48) including sensitivity in Tumor Control Probability (TCP) and Normal Tissue Complication Probability (NTCP) (49). A recent review article agrees multiple endpoints are needed in assessing contour quality, and clinical validation of meaningful TCP/NTCP endpoints will guide meaningful contour deviations (50). In dose escalated TMI/TMLI maximum dose may not be a critical evaluation datapoint. Instead, volume-based metrics such as V10, V50, and V80 may be more useful to identify quality treatment plans. Our results demonstrate that DVH-based metrics are not closely related to Dice, HD-95, or HD<1mm in OARs, but in general HD<1mm > 60% and Dice>90% led to consistent DVHs. In PTVs, the scenario is much different, and we consistently observed PTV-DVH differences >0.30 across all dose levels even with very high Dice scores. Sufficient target delineation is an essential requirement of conformal radiation to ensure disease control and reduce the possibility of underdosing the target (51). Over-contouring PTV results in a larger treatment volume in normal tissue, which is very familiar to conventional TBI regimens but may not be appropriate for TMI and TMLI.

## 5 Conclusions

Utilization of auto-segmentation for TMI and TMLI treatment planning presents a breakthrough for clinical efficiency in implementation of TMI/TMLI treatments. Efficiency gains of 80-90% are possible in >20 structures including PTVs and OARs.

## Data availability statement

The data supporting the conclusions of this article will be made available by the corresponding author upon request with permission from City of Hope National Medical Center and Medical Mind, Inc. where applicable.

## Author contributions

WW collected data, wrote scientific code, performed analysis, and designed the study. AL developed the TMI model and co-developed the scientific design of the study. KQ, CH, and SH provided expertise in the area of TMI/TMLI and contributed to the article text. All authors contributed to the article and approved the submitted version.

## Conflict of interest

AL, KQ, CH, and TW have a research collaboration with Medical Mind, Inc.

The remaining author declares that the research was conducted in the absence of any commercial or financial relationships that could be construed as a potential conflict of interest.

## Publisher’s note

All claims expressed in this article are solely those of the authors and do not necessarily represent those of their affiliated organizations, or those of the publisher, the editors and the reviewers. Any product that may be evaluated in this article, or claim that may be made by its manufacturer, is not guaranteed or endorsed by the publisher.
